# Prevalence of Anxiety, Depression, and Distress and Their Association With Problems Encountered by Advanced Cancer Patients in a Tertiary Hospital in Saudi Arabia

**DOI:** 10.7759/cureus.66219

**Published:** 2024-08-05

**Authors:** Abdulaziz Bakhsh, Gassan Abudari, Saud Alhaidar, Saad Shamsy, Ahlam Alqahtani, Rania Haddadi, Maiadh Almsaud, Steven Callaghan, Fawad Ahmad

**Affiliations:** 1 Oncology Center, King Faisal Specialist Hospital and Research Centre, Riyadh, SAU; 2 Oncology Nursing, King Faisal Specialist Hospital and Research Centre, Riyadh, SAU; 3 Department of Social Services, King Faisal Specialist Hospital and Research Centre, Riyadh, SAU

**Keywords:** saudi arabia, depression, anxiety, distress, cancer patients, palliative care

## Abstract

Background: Patients with advanced cancer often suffer from significant psychological distress, anxiety, and depression, which can profoundly influence their quality of life. This study aimed to evaluate the prevalence and severity of these psychological factors in advanced cancer patients. Additionally, it sought to identify related psychosocial, practical, emotional, and physical problems and their association with the psychological factors. Furthermore, this study provides interventions and strategies to help mitigate the psychological burden experienced by these patients.

Methods: A cross-sectional survey involving 180 patients with advanced cancer was conducted at a tertiary hospital in Saudi Arabia. Participants were assessed using the Distress Thermometer (DT) and the Hospital Anxiety and Depression Scale (HADS). Data analysis included descriptive statistics, chi-square tests for categorical variables, and multivariate regression to explore the factors associated with distress, anxiety, and depression.

Results: The prevalence of distress, anxiety, and depression among patients was 40.6%, 46.1%, and 52.2%, respectively. Patients who experienced 'changes in urination' which is an item in DT had a 2.86 times higher risk of developing distress. Patients experiencing sadness (item in DT) and fatigue (item in DT) were at a 3.91 and 2.29 times higher risk of developing anxiety, respectively. Practical problems, such as childcare and treatment decisions, emotional problems, and physical problems, such as appearance, bathing/dressing, and eating difficulties, were significantly associated with distress. There was no significant association between patients' demographics and psychological factors.

Conclusion: The findings underscore the complex interplay of psychosocial, practical, emotional, and physical problems faced by advanced cancer patients receiving palliative care. These patients exhibit a high percentage of distress, anxiety, and depression. Addressing these multifaceted problems through targeted psychological and social interventions can significantly enhance the overall care and quality of life for this vulnerable population. This study advocates routine psychological screenings and tailored interventions to mitigate the psychological burden in this group.

## Introduction

Cancer in advanced stages poses significant physical and psychosocial challenges for patients. As patients struggle to realize the reality of diagnosis and the complexities of treatment, they often experience myriad emotional, social, and practical difficulties that can profoundly impact their quality of life [1,2]. Psychological distress, anxiety, and depression are particularly prominent among these challenges [3,4].

The prevalence of these psychological issues in cancer patients varies widely across studies, with distress reported in 20-40% of patients, anxiety in 10-30%, and depression in 20-50% [5-10]). These variations may be attributed to a multitude of factors, such as cancer types, stages, treatment modalities, age and gender [11,12]. Therefore, psychological conditions are markedly higher in cancer patients than in the general population, underscoring the need for targeted psychosocial interventions [13].

The palliative care approach philosophy focuses on providing holistic patient-centered care. Therefore, it is crucial to address patients’ psychosocial needs. Enhancing the overall quality of life of patients with advanced cancer requires integrating psychological support with physical symptom management [14]. However, despite the recognized importance of psychosocial aspects in palliative care, many patients' needs remain unmet, often due to inadequate screening, lack of professional staff, or insufficient resources [1-15]. Several studies have shown that anxiety and depression are frequently undiagnosed and untreated in patients with advanced cancer who receive palliative care [2,8,16]. One study conducted in Saudi Arabia found that 42% of cancer patients had anxiety, 28% had depression, and only 15% of patients were prescribed medication to treat both symptoms [11].

Palliative care services have evolved in Saudi Arabia, where this study was conducted. However, the accessibility of the services is still a challenge, as most palliative centers are located in major cities [17]. Furthermore, research on the psychosocial aspects of care for patients with advanced cancer remains limited. Understanding the psychosocial characteristics and burden of symptoms in this specific population is crucial for developing culturally sensitive and effective psychosocial interventions.

This study aimed to systematically assess the prevalence and severity of distress, anxiety, and depression in patients with advanced cancer. Additionally, this research identified the specific psychosocial, emotional, practical, and physical problems encountered by these patients and explored how these problems influence their psychological state. The ultimate goal is to enhance the understanding of the holistic needs of patients with advanced cancer to better tailor palliative care interventions that address their unique challenges.

## Materials and methods

This cross-sectional survey was conducted to investigate the psychological, social, physical and practical problems of patients with advanced cancer as well as the prevalence of anxiety, depression and distress for these patients. The study was conducted at a tertiary hospital, King Faisal Specialist Hospital and Research Center in Riyadh, Saudi Arabia, where patients were either receiving palliative care as their primary treatment or seen on a consultation basis. Participants were recruited from both inpatient wards and palliative care clinics between January 2024 and April 2024.

For inclusion criteria, patients were eligible if they were older than 18 years of age, received palliative care service, and were capable of providing informed consent. Any patient diagnosis with cancer and not eligible for a curative treatment was considered as advanced cancer. 

For exclusion criteria, patients were excluded if they had Impairments in cognitive or language abilities that could hinder informed consent or questionnaire comprehension. Furthermore, the presence of acute physical or psychological distress that may exacerbate the patient's burden when participating in the study or patients with pre-existing psychological disorders such as anxiety disorder or depression existing before diagnosis of cancer.

Data collection instruments

Socio-Demographic and Clinical Data

Socio-demographic data for each participant included age, gender, marital status, education, and employment. Clinical data including diagnosis, code status, and active oncology treatment were collected and recorded.

Distress Thermometer (DT)

DT is a tool for cancer patients to rate their distress on a scale from 0 to 10. It includes a list of problems to identify the specific sources of distress. A score of 4-6 may indicate possible distress, whereas a score of 7-10 signifies definite distress [18]. Another study found that a DT cut-off score of 6 or above is an appropriate point for identifying patients with a significant level of distress [19]. The Problem List in DT covers 38 problems grouped into five domains: practical, physical, emotional, family, and spiritual concerns. The Validated Arabic version of the DT scale was used to assess distress levels [20]. No modifications were made to the original Arabic DT.

The Hospital Anxiety and Depression Scale (HADS)

HADS was initially introduced as a tool to detect the state of depression and anxiety in non-psychiatric patients [21]. Recognized as an effective screening tool for anxiety and depression, it has been used extensively in cancer research and practice. On both the anxiety (HADS-A) and depression (HADS-D) subscales, a cutoff score of 8 was determined for questionable cases and 11 for conclusive cases in the initial study. Nonetheless, the majority of further research has determined that 8 is the ideal cutoff for both HADS-A and HADS-D [22,23]. The translated and validated Arabic version of the HADS was utilized [24].

Ethical considerations

The study received approval from the King Faisal Specialist Hospital and Research Centre Institutional Review Board (approval number: RAC# 2231075). All participants were informed about the study objectives, and informed consent was obtained prior to participation. The participants were assured of their right to withdraw from the study at any point without any negative repercussions. Confidentiality and anonymity of the participants' responses were maintained throughout the research process.

Statistical analysis

Descriptive statistics were given as numbers and percentages (%) for all categorical variables, while mean and standard deviation were used to describe continuous variables. The relationship between the problems encountered and the levels of distress has been conducted using the Chi-square test. Significant results were tested in the multivariate regression analysis to determine the significant independent risk factors associated with distress. Also, univariate analyses were performed to find out the link between distress, anxiety, and depression in terms of the socio-demographic characteristics of the patients. Values were considered significant with a p-value of less than 0.05. The data were analyzed using the software program Statistical Packages for Software Sciences (SPSS) version 26 (IBM Corp., Armonk, NY, USA).

## Results

This study recruited 180 patients with advanced cancer with no curative treatment and followed by the palliative care team as a primary service or consultation. As described in Table 1, 28.9% were over 60 years old. More than half were females (55%), and most were married (68.3%). Patients who were bachelor's degree holders constituted 42.8%. Approximately 60.6% were unemployed. Most patients received treatment in outpatient palliative care (82.2%). The prevalence of patients who were currently receiving treatment was 39.4%.

**Table 1:** Socio-demographic characteristics of the patients (n=180)

Study data	N (%)
Age group	
<40 years	36 (20.0%)
40 – 50 years	51 (28.3%)
51 – 60 years	41 (22.8%)
>60 years	52 (28.9%)
Gender	
Male	81 (45.0%)
Female	99 (55.0%)
Marital status	
Single	23 (12.8%)
Married	123 (68.3%)
Divorced	18 (10.0%)
Widowed	16 (08.9%)
Educational level	
Uneducated	19 (10.6%)
Primary or secondary	39 (21.7%)
High school	45 (25.0%)
Bachelor's degree	77 (42.8%)
Employment status	
Unemployed	109 (60.6%)
Government employee	47 (26.1%)
Private employee	24 (13.3%)
Location	
Outpatient palliative care	148 (82.2%)
Inpatient palliative care	12 (06.7%)
Consultation	20 (11.1%)
Active treatment	
No	109 (60.6%)
Yes	71 (39.4%)

The most commonly diagnosed cancer was gastrointestinal cancer (26.7%), followed by breast cancer (23.3%) and hematological cancer (10.6%). The details of the HADS questionnaire are further discussed in Table 2, representing means and standard deviations for each response to every domain.

**Table 2:** Details of the Hospital Anxiety and Depression Scale (HADS) questionnaire (n=180) Response has a category range from 0 to 3 points.

Items	Mean ± SD
Anxiety items	
1. I feel tense	1.13 ± 0.91
2. I get a sort of frightened feeling as if something awful is about to happen	0.96 ± 0.95
3. I can sit at ease and feel relaxed	1.36 ± 0.94
4. Worrying thoughts go through my mind	1.19 ± 1.07
5. I get a sort of frightened feeling like butterflies in the stocmach	0.84 ± 0.91
6. I feel restless as if I have to be on the move	1.28 ± 1.06
7. I get sudden feelings of panic	0.88 ± 0.91
Depression items	
1. I still enjoy the things I used to enjoy	1.24 ± 0.99
2. I can laugh and see the funny side of things	0.97 ± 0.92
3. I feel cheerful	1.12 ± 0.92
4. I feel as if I am slowed down	1.58 ± 0.96
5. I have lost interest in my appearance	0.96 ± 1.09
6. I look forward with enjoyment to things	1.00 ± 0.94
7. I can enjoy a good book or radio or TV program	1.18 ± 1.15

Regarding the descriptive statistics of anxiety and depression (Table 3), it was observed that the mean score of anxiety and depression according to the HADS criteria were 7.64 and 8.05, respectively. 28.3% and 26.7% were considered to be in abnormal levels of anxiety and depression. Patients who were deemed anxious and depressed were 46.1% and 52.2%, respectively. Regarding distress, the mean score of the DT questionnaire was 4.96, and the prevalence of distress among cancer patients was 40.6%.

**Table 3:** Assessment of anxiety, depression, and distress using the Hospital Anxiety and Depression Scale (HADS) and Distress Thermometer (DT) questionnaire (n=180)

Variables	N (%)
Anxiety score (mean ± SD)	7.64 ± 4.51
Normal (score 0 – 7)	97 (53.9%)
Borderline (score 8 – 10)	32 (17.8%)
Abnormal (score 11 – 21)	51 (28.3%)
Level of anxiety	
Anxious (score ≥8)	83 (46.1%)
Not anxious (score <8)	97 (53.9%)
Depression score (mean ± SD)	8.05 ± 4.48
Normal (score 0 – 7)	86 (47.8%)
Borderline (score 8 – 10)	46 (25.6%)
Abnormal (score 11 – 21)	48 (26.7%)
Level of depression	
Depressed (score ≥8)	94 (52.2%)
Not depressed (score <8)	86 (47.8%)
DT score (mean ± SD)	4.96 ± 2.72
Level of distress	
Distressed (score ≥6)	73 (40.6%)
Not distressed (score<6)	107 (59.4%)

The most common practical problem encountered was transportation (26.1%), followed by insurance/financial (23.9%) and treatment decisions (20%). Family health issues are the most common family problems encountered by the patients, followed by dealing with children (18.3%) and the ability to have children (15.6%). Loss of interest in usual activities was the patients' most frequently encountered emotional problem, followed by nervousness (41.7%) and worry (35.6%). The top five physical problems encountered by the patients were pain (67.2%), fatigue (63.9%), constipation (54.4%), dry itchy skin (41.7%) and eating (41.7%).

When measuring the effect of encountered problems to the distress levels of the patients (Table 4), it was revealed that the prevalence of distress was significantly more common among patients who encountered problems related to childcare (p<0.001), housing (p=0.005), insurance/financial (0.007), treatment decisions (p=0.001), dealing with a partner (p=0.010), ability to have children (p=0.005), fear (p<0.001), sadness (p=0.001), worry (p<0.001), loss of interest in usual activities (p=0.003), appearance (p=0.009), bathing/dressing (p=0.014), changes in urination (p<0.001), diarrhea (p=0.038), feeling swollen (p=0.035), getting around (p=0.005), nausea (p=0.036), sexual (p=0.033), dry or itchy skin (p=0.015) and substance abuse (p=0.018).

**Table 4:** Relationship between the problems list encountered and distress level (n=180) § P-value has been calculated using Chi-square test. ** Significant at p<0.05 level.

Problem list	Distressed level		P-value ^§^
	Distressed N (%) ^(n=73)^	Not distressed N (%) ^(n=107)^	
Practical problems			
Childcare	18 (24.7%)	06 (05.6%)	<0.001 **
Housing	22 (30.1%)	14 (13.1%)	0.005 **
Insurance/Financial	25 (34.2%)	18 (16.8%)	0.007 **
Transportation	24 (32.9%)	23 (21.5%)	0.088
Work/School	16 (21.9%)	13 (12.1%)	0.080
Treatment Decisions	25 (34.2%)	15 (14.0%)	0.001 **
Family problems			
Dealing with children	17 (23.3%)	16 (15.0%)	0.156
Dealing with partner	17 (23.3%)	10 (09.3%)	0.010 **
Ability to have children	18 (24.7%)	10 (09.3%)	0.005 **
Family health issues	21 (28.8%)	22 (20.6%)	0.205
Emotional problems			
Fear	33 (45.2%)	19 (17.8%)	<0.001 **
Nervousness	36 (49.3%)	39 (36.4%)	0.086
Sadness	36 (49.3%)	26 (24.3%)	0.001 **
Worry	41 (56.2%)	23 (21.5%)	<0.001 **
Loss of interest in usual activities	44 (60.3%)	40 (37.4%)	0.003 **
Physical problems			
Appearance	20 (27.4%)	13 (12.1%)	0.009 **
Bathing/Dressing	22 (30.1%)	16 (15.0%)	0.014 **
Breathing	24 (32.9%)	39 (36.4%)	0.622
Changes in urination	34 (46.6%)	21 (19.6%)	<0.001 **
Constipation	36 (49.3%)	62 (57.9%)	0.254
Diarrhea	21 (28.8%)	17 (15.9%)	0.038 **
Eating	36 (49.3%)	39 (36.4%)	0.086
Fatigue	52 (71.2%)	63 (58.9%)	0.090
Felling swollen	36 (49.3%)	36 (33.6%)	0.035 **
Fever	19 (26.0%)	11 (10.3%)	0.005 **
Getting around	27 (37.0%)	35 (32.7%)	0.553
Indigestion	29 (39.7%)	33 (30.8%)	0.218
Memory concentration	28 (38.4%)	30 (28.0%)	0.146
Mouth sore	25 (34.2%)	23 (21.5%)	0.058
Nausea	33 (45.2%)	32 (29.9%)	0.036 **
Nose dry or congested	27 (37.0%)	29 (27.1%)	0.160
Pain	53 (72.6%)	68 (63.6%)	0.204
Sexual	15 (20.5%)	10 (09.3%)	0.033 **
Skin dry or itchy	40 (54.8%)	39 (36.4%)	0.015 **
Sleep	13 (17.8%)	09 (08.4%)	0.059
Substance abuse	13 (17.8%)	07 (06.5%)	0.018 **
Tingling in hand and feet	30 (41.1%)	36 (33.6%)	0.308

In a univariate analysis of the factors that influence distress (Table 5), it was revealed that 52.1% and 39.7% of the distressed patients had concurrent anxiety (p<0.001) and depression (p=0.001). No significant relationships were observed between distress and the socio-demographic variables of the patients (p>0.05).

**Table 5:** Univariate analysis of the factors that influence distress (n=180) § P-value has been calculated using Chi-square test. ** Significant at p<0.05 level.

Factor	Distressed level		P-value ^§^
	Distressed N (%) ^(n=73)^	Not distressed N (%) ^(n=107)^	
Age group			
≤50 years	40 (54.8%)	47 (43.9%)	0.152
>50 years	33 (45.2%)	60 (56.1%)	
Gender			
Male	33 (45.2%)	48 (44.9%)	0.963
Female	40 (54.8%)	59 (55.1%)	
Marital status			
Unmarried	19 (26.0%)	38 (35.5%)	0.179
Married	54 (74.0%)	69 (64.5%)	
Educational level			
High school or below	40 (54.8%)	63 (58.9%)	0.587
Bachelor's degree	33 (45.2%)	44 (41.1%)	
Employment status			
Unemployed	43 (58.9%)	66 (61.7%)	0.708
Employed	30 (41.1%)	41 (38.3%)	
Active treatment			
No	48 (65.8%)	61 (57.0%)	0.239
Yes	25 (34.2%)	46 (43.0%)	
Level of anxiety			
Anxious	54 (74.0%)	29 (27.1%)	<0.001 **
Not Anxious	19 (26.0%)	78 (72.9%)	
Level of depression			
Depressed	49 (67.1%)	45 (42.1%)	0.001 **
Not depressed	24 (32.9%)	62 (57.9%)	

In Table 6, it was revealed that all socio-demographic data did not significantly influence the presence of both anxiety and depression (all p>0.05).

**Table 6:** Univariate analysis of the factors that influence anxiety and depression (n=180) § P-value has been calculated using Chi-square test.

Factor	Anxiety level		P-value ^§^	Depression level		P-value ^§^
	Anxious N (%) ^(n=83)^	Not Anxious N (%) ^(n=97)^		Depressed N (%) ^(n=94)^	Not depressed N (%) ^(n=86)^	
Age group						
≤50 years	43 (51.8%)	44 (45.4%)	0.388	48 (51.1%)	39 (45.3%)	0.443
>50 years	40 (48.2%)	53 (54.6%)		46 (48.9%)	47 (54.7%)	
Gender						
Male	31 (37.3%)	50 (51.5%)	0.056	37 (39.4%)	44 (51.2%)	0.112
Female	52 (62.7%)	47 (48.5%)		57 (60.6%)	42 (48.8%)	
Marital status						
Unmarried	28 (33.7%)	29 (29.9%)	0.581	31 (33.0%)	26 (30.2%)	0.692
Married	55 (66.3%)	68 (70.1%)		63 (67.0%)	60 (69.8%)	
Educational level						
High school or below	52 (62.7%)	51 (62.6%)	0.173	58 (61.7%)	45 (52.3%)	0.204
Bachelor's degree	31 (37.3%)	46 (47.4%)		36 (38.3%)	41 (47.7%)	
Employment status						
Unemployed	54 (65.1%)	55 (56.7%)	0.253	59 (62.8%)	50 (58.1%)	0.526
Employed	29 (34.9%)	42 (43.3%)		35 (37.2%)	36 (41.9%)	
Active treatment						
No	51 (61.4%)	58 (59.8%)	0.821	59 (62.8%)	50 (58.1%)	0.526
Yes	32 (38.6%)	39 (40.2%)		35 (37.2%)	36 (41.9%)	

When conducting a multivariate regression model (Table 7), it was revealed that patients who encountered changes in urination for the past week were predicted to increase the risk of developing distress by at least 2.86 times higher (AOR=2.865; 95% CI=1.292 - 6.352; p=0.010). No significant effects were observed with the rest of the problems encountered for the past week in terms of distress after adjustment to a regression model (p>0.05). Further, patients who encountered sadness for the past week were predicted to increase the risk of developing anxiety by at least 3.91-fold higher (AOR=3.912; 95% CI=1.479 - 10.351; p=0.006). Also, patients who encountered fatigue for the past week were at increased risk of developing anxiety by at least 2.29 times higher (AOR=2.291; 95% CI=1.004 - 5.225; p=0.049). In contrast, this study found no significant differences between the significant problems encountered for the past week in univariate analysis and the developing depression after adjustment to a regression model (all p>0.05).

**Table 7:** Multivariate regression analysis to determine the significant independent risk factors associated with distress, anxiety, and depression (n=180) AOR – Adjusted Odds Ratio; CI – Confidence Interval. ** Significant at p<0.05 level.

Distressed	AOR	95% CI	P-value
Practical problems			
Childcare	2.352	0.628 – 8.816	0.204
Housing	1.197	0.453 – 3.167	0.717
Insurance/Financial	1.590	0.632 – 3.997	0.324
Treatment Decisions	1.837	0.695 – 4.855	0.220
Family problems			
Dealing with partner	1.146	0.359 – 3.658	0.818
Ability to have children	1.295	0.425 – 3.945	0.650
Emotional problems			
Fear	2.036	0.713 – 5.812	0.184
Sadness	0.588	0.182 – 1.899	0.375
Worry	2.005	0.761 – 5.281	0.159
Loss of interest in usual activities	1.516	0.665 – 3.455	0.322
Physical problems			
Appearance	0.556	0.165 – 1.869	0.343
Bathing/Dressing	1.495	0.494 – 4.521	0.476
Changes in urination	2.865	1.292 – 6.352	0.010 **
Diarrhea	1.114	0.422 – 2.937	0.828
Felling swollen	0.998	0.431 – 2.311	0.996
Fever	1.917	0.624 – 5.890	0.256
Nausea	1.029	0.432 – 2.450	0.949
Sexual	0.704	0.202 – 2.452	0.581
Substance abuse	0.555	0.121 – 2.534	0.447
Anxiety			
Emotional problems			
Fear	1.214	0.455 – 3.237	0.698
Nervousness	0.905	0.397 – 2.064	0.813
Sadness	3.912	1.479 – 10.351	0.006 **
Worry	1.780	0.725 – 4.367	0.208
Loss of interest in usual activities	1.275	0.551 – 2.949	0.571
Physical problems			
Bathing/Dressing	0.712	0.266 – 1.904	0.498
Eating	1.062	0.488 – 2.313	0.880
Fatigue	2.291	1.004 – 5.225	0.049 **
Felling swollen	1.134	0.493 – 2.609	0.767
Fever	1.370	0.491 – 3.821	0.547
Getting around	1.128	0.506 – 2.512	0.768
Memory concentration	1.478	0.652 – 3.352	0.350
Skin dry or itchy	1.727	0.839 – 3.554	0.138
Depression			
Emotional problems			
Sadness	0.874	0.368 – 2.077	0.761
Worry	1.188	0.504 – 2.801	0.694
Loss of interest in usual activities	1.379	0.653 – 2.911	0.400
Physical problems			
Appearance	2.713	0.935 – 7.876	0.066
Bathing/Dressing	1.249	0.494 – 3.157	0.638
Eating	1.616	0.823 – 3.173	0.163
Getting around	1.737	0.846 – 3.566	0.132
Indigestion	0.722	0.356 – 1.463	0.366

## Discussion

This study investigated the psychosocial well-being of patients with advanced cancer receiving palliative care. The findings of this study provide additional insights into the psychological well-being of this group of patients. Studies suggest that psychological disorders are prevalent in patients with advanced cancer seeking palliative care, who have an increased burden of symptoms. Hence, the present study also explored the effect of several issues, including practical, physical, social, and emotional, on the psychological characteristics of cancer patients.

Prevalence and influencing factors of distress

Distress is a detrimental factor that affects patients with advanced cancer. According to our results, more than 40% of patients were considered distressed, without significant differences in terms of patients' socio-demographic profiles. This is consistent with the study of Funk-Lawler et al. (2020), who reported that nearly half of the patients were severely distressed, but there was no significant association between the total distress score and demographic and illness-related characteristics [25]. However, several studies found demographic correlations with distress levels, such as younger age, gender and type of cancer [19,26,27]. The patients in this study reported a wide range of distressing issues that were significantly influenced by practical, family issues, emotional, and physical factors. These issues include everyday practicalities, such as childcare and personal hygiene, as well as deep personal concerns involving family dynamics and decision-making regarding treatment. Specific problems, such as difficulties with bathing, urination, and managing childcare, not only compounded their physical discomfort, but also heightened their emotional distress. This finding matches previous studies that have highlighted the link between distress and emotional, physical, and practical issues [28,29]. However, in this study, patients frequently reported experiencing physical problems such as pain and fatigue, which occurred with the highest frequency, along with common issues that cause distress, such as breathing difficulties. Despite their prevalence, these physical symptoms did not show a significant correlation with levels of distress among patients. This finding suggests that, while these physical issues are common and impactful, they do not necessarily predict or directly contribute to the emotional and psychological distress experienced by patients. Research on the relationship between physical problems and psychological distress in palliative care presents mixed findings. Some studies have identified a significant correlation; conversely, other studies have not found a consistent correlation, indicating that the link between physical symptoms and distress may be influenced by various other factors including psychological resilience, social support, and the effectiveness of symptom management strategies [30,31]. This highlights the complex nature of distress in palliative care, in which not all prevalent symptoms directly influence patients' psychological well-being.

Prevalence and influencing factors anxiety

Anxiety is another detrimental factor influencing patients with advanced cancer. In this study, nearly half of the cancer patients showed symptoms of anxiety. However, comparing the anxiety levels in relation to the patients’ demographic data, this study found no significant relationship between each demographic factor and anxiety. Several studies have documented that anxiety is prevalent among cancer patients [8-32]. Anxiety in patients with advanced cancer is influenced by various factors, such as gender, younger age, high burden of physical symptoms, and lack of spirituality [33,34]. In this study, it was observed that patients experiencing emotional problems, particularly sadness, along with physical challenges such as fatigue, demonstrated higher levels of anxiety. Additionally, although to a lesser extent, problems with memory concentration and skin dryness or itchiness were associated with increased anxiety levels. One study found that anxiety levels were heightened by emotional distress in patients with advanced cancer [35]. Furthermore, fatigue was associated with greater anxiety, depressive symptoms, and a worse quality of life among cancer patients [36]. These results affirm the need for holistic care approaches that address not only the physical, but also the emotional and psychosocial aspects of cancer treatment. This approach ensures that patients receive well-rounded care that considers their overall well-being, not just their medical condition. Therefore, understanding these interdependencies is crucial for developing targeted interventions aimed at reducing anxiety and improving the overall quality of life of patients with advanced cancer.

Prevalence and influencing factors of depression

One of the prominent findings of this study was that more than half of the cancer patients showed symptoms of depression. Various papers reported that depression is common among cancer patients, ranging from 24% to 54% [8,25,37-39]. A study conducted in Saudi Arabia found no significant correlation between depression and age [39]. This agrees with our results, as we found no significant relationship between depression and age, gender, marital status, education, employment status, and active treatment. However, a few previous studies have found an association between depression and patients' demographics such as gender, age, and educational level [16-40]. Depression in advanced cancer is significantly correlated with physical symptoms such as pain, fatigue, drowsiness, and constipation [41,42]. In this study, depression was associated with emotional problems, such as sadness and loss of interest, as well as physical problems, such as appearance, bathing, and indigestion. However, common physical symptoms such as pain and fatigue were not associated with depression among the investigated population. In contrast, previous studies have shown a significant correlation between fatigue as well as pain with depression in advanced cancer [40-43]. The absence of an association between common symptoms and depression is probably because this study did not investigate the intensity of these symptoms or the social support system surrounding the patients, which can provide psychological resilience to alleviate the impact of these symptoms [44]. Furthermore, this finding is probably related to the fact that physical symptoms are commonly prioritized in palliative care assessment and management [45]. It is essential to prioritize depression and psychosocial symptom assessments with the same level of attention as physical symptom assessments in the management of patients with advanced cancer. 

Practical problems encountered

Practical problems were only associated with patient distress without any effect on anxiety and depression. Transportation was the most prominent practical problem experienced by the patients. In this study, transportation was highly reported because of the location of the tertiary hospital that provides services to patients from around Saudi Arabia. Previous studies have reported that this problem is common, especially in patients living in rural areas [46,47]. Treatment decision was another frequent problem encountered by patients. Patients with advanced cancer have high burden of decision-making due to risk, uncertainty, and conflicting emotions [48]. The complexity of treatment decisions for patients with advanced cancer is immense. These decisions often involve weighing the benefits and risks of the various treatment options. Each option carries its own set of potential outcomes and side effects, which can add more psychological and emotional burden [48]. Patients often struggle to understand the intricate details of treatment options. The medical jargon used in discussions and the sheer volume of information can be overwhelming [49,50]. Moreover, patients frequently experience conflicting emotions and uncertainty about their choices to pursue aggressive treatment [51]. Implementing shared decision-making where patients and healthcare providers collaboratively make treatment decisions was found to decrease distress [52].

Family problems encountered

Family problems were only associated with distress without any effects on anxiety and depression. The two main significant problems were “relation with partner” and child care. Dealing with partner in advanced cancer poses various challenges that impact the partners’ relationship. Studies have shown that partners facing advanced cancer experience uncertainty about the future, changes in intimacy, and role adjustments [53]. Additionally, issues related to sexual health can affect feelings of closeness between partners [54]. Furthermore, family involvement in the care and decision-making can lead sometimes to conflicts and burdens [55]. It is crucial for healthcare providers to assess any family conflict based on family dynamics [56]. Involvement of members of an interdisciplinary team such as social workers and psychotherapists was found to be effective in improving relations with partners [55-57].

Physical problems encountered

This study underscores the substantial physical symptom burden experienced by patients with advanced cancer who receive palliative care. The most commonly reported physical problems were pain (67.2%), fatigue (63.9%), constipation (54.4%), skin dryness or itchiness (43.9%), and eating difficulties (41.7%). This finding aligns with previous studies that identified pain, fatigue, and gastrointestinal symptoms as the predominant cancer in advanced cancer [10]. However, pain and constipation were not associated with distress, anxiety, or depression in this study, whereas fatigue was only associated with anxiety. Physical problems, such as bathing and dressing, appearance and eating contribute to distress, anxiety, and depression. One study reported that about 36% of cancer patients reported having problems with their appearance, and this issue was more common among younger patients and females, and those patients showed higher levels of depression, anxiety, and distress [58]. Patients with advanced cancer often experience impairments in daily activities and independence in daily functioning due to the progression of their disease and treatment-related toxicities [59]. Adequate palliative rehabilitation has shown a positive impact on performing daily living activities in patients with advanced cancer, particularly those who have lost their grooming abilities [60]. Eating-related distress (ERD) is a significant concern among patients with advanced cancer, affecting their psychological well-being and quality of life. Studies have shown that impaired nutrition is associated with greater depression and lower quality of life in older adults with advanced cancer [61]. Additionally, malnutrition in advanced cancer patients is common and can lead to emotional and social impacts, affecting both patients and their family caregivers [62]. Eating distress in advanced cancer requires multidimensional nutritional interventions [63]. One of the effective interventions to improve eating-related distress is focusing on family-centered nutritional support [64]. The most distressing problem encountered in this population was a change in urination. Urinary dysfunction is a prevalent issue among advanced cancer patients, impacting their quality of life [65]. Dysuria and hematuria are the most common urinary symptoms and are usually caused by tumor location, opioid-related or oncology treatment such as radiation, chemotherapy, or surgery [66-68]. Several palliative interventions including, medications, behavioral therapy, and minimal invasive therapy can relieve patients' symptoms [69].

These findings have important implications for palliative care practice. They emphasize the need for comprehensive symptom assessment and management, focusing not only on the most common symptoms such as pain, shortness of breath, and constipation, but also on those that might be overlooked yet significantly impact psychological well-being, such as urinary changes, eating-distress and carrying out daily living activities. However, the lack of consistent associations between many physical symptoms and psychological outcomes does not diminish their importance. Rather, it highlights the need for personalized, patient-centered care.

Clinical implications

The significant levels of distress, anxiety, and depression identified in this study highlight the need for routine psychological screening in palliative care settings. This can facilitate early intervention and potentially improve patient outcomes. The findings also suggest that healthcare systems should allocate more resources to integrative care services to ensure that psychological support is accessible in palliative care settings. Moreover, enhanced training programs for healthcare providers could equip them with skills to effectively manage the complex psychosocial needs of cancer patients effectively.

Limitations

The cross-sectional design of this study limits the ability to establish causality between psychosocial characteristics and patient outcomes. Conducted at a single tertiary hospital in Saudi Arabia, the findings may not be generalizable to other settings. Additionally, reliance on self-reported measures to assess distress, anxiety, and depression can introduce response biases. The exclusion of patients with cognitive impairments or severe psychological distress may also result in the underestimation of the prevalence and intensity of psychosocial issues, potentially skewing the findings towards less severe cases.

## Conclusions

This study highlights the substantial psychological challenges faced by advanced cancer patients receiving palliative care in a tertiary hospital in Saudi Arabia. The significant prevalence of distress, anxiety, and depression underscores the critical need for comprehensive care strategies that address both physical and psychological burdens. Practical issues like treatment decisions and transportation, along with emotional struggles such as sadness and worry, were significantly linked to higher levels of distress and anxiety.

Despite the high occurrence of physical symptoms like pain and fatigue, these were not consistently associated with psychological outcomes, indicating the complexity of the relationship between physical and mental health in palliative care. This finding emphasizes the need for personalized, patient-centered care approaches that address a broader spectrum of symptoms impacting psychological well-being. Routine psychological screenings and tailored interventions are crucial to alleviate the psychological burden among advanced cancer patients.

The study advocates for more resources to be allocated to integrative care services, ensuring psychological support is accessible within palliative care frameworks. Enhanced training programs for healthcare providers are essential to manage the multifaceted psychosocial, physical and practical needs of cancer patients effectively. While the cross-sectional design limits causality and generalizability, the insights gained underscore the importance of a holistic approach in palliative care, which can significantly enhance the quality of life for patients with advanced cancer.

## References

[1] Jordan K, Aapro M, Kaasa S (2018). European Society for Medical Oncology (ESMO) position paper on supportive and palliative care. Ann Oncol.

[2] Kissane DW (2014). Unrecognised and untreated depression in cancer care. Lancet Psychiatry.

[3] Linden W, Vodermaier A, Mackenzie R, Greig D (2012). Anxiety and depression after cancer diagnosis: prevalence rates by cancer type, gender, and age. J Affect Disord.

[4] Zabora J, BrintzenhofeSzoc K, Curbow B, Hooker C, Piantadosi S (2001). The prevalence of psychological distress by cancer site. Psychooncology.

[5] Bužgová R, Jarošová D, Hajnová E (2015). Assessing anxiety and depression with respect to the quality of life in cancer inpatients receiving palliative care. Eur J Oncol Nurs.

[6] Delgado-Guay M, Parsons HA, Li Z, Palmer JL, Bruera E (2009). Symptom distress in advanced cancer patients with anxiety and depression in the palliative care setting. Support Care Cancer.

[7] He Y, Pang Y, Su Z (2022). Symptom burden, psychological distress, and symptom management status in hospitalized patients with advanced cancer: a multicenter study in China. ESMO Open.

[8] Sewtz C, Muscheites W, Grosse-Thie C (2021). Longitudinal observation of anxiety and depression among palliative care cancer patients. Ann Palliat Med.

[9] Smith EM, Gomm SA, Dickens CM (2003). Assessing the independent contribution to quality of life from anxiety and depression in patients with advanced cancer. Palliat Med.

[10] Van Lancker A, Velghe A, Van Hecke A (2014). Prevalence of symptoms in older cancer patients receiving palliative care: a systematic review and meta-analysis. J Pain Symptom Manage.

[11] Naser AY, Hameed AN, Mustafa N, Alwafi H, Dahmash EZ, Alyami HS, Khalil H (2021). Depression and anxiety in patients with cancer: a cross-sectional study. Front Psychol.

[12] Pitman A, Suleman S, Hyde N, Hodgkiss A (2018). Depression and anxiety in patients with cancer. BMJ.

[13] Hinz A, Krauss O, Hauss JP, Höckel M, Kortmann RD, Stolzenburg JU, Schwarz R (2010). Anxiety and depression in cancer patients compared with the general population. Eur J Cancer Care (Engl).

[14] Rohrmoser A, Preisler M, Bär K, Letsch A, Goerling U (2017). Early integration of palliative/supportive cancer care-healthcare professionals' perspectives on the support needs of cancer patients and their caregivers across the cancer treatment trajectory. Support Care Cancer.

[15] Hong J, Song Y, Liu J, Wang W, Wang W (2014). Perception and fulfillment of cancer patients' nursing professional social support needs: from the health care personnel point of view. Support Care Cancer.

[16] Obispo-Portero B, Cruz-Castellanos P, Jiménez-Fonseca P (2022). Anxiety and depression in patients with advanced cancer during the COVID-19 pandemic. Support Care Cancer.

[17] Alshammary S, Altamimi I, Alhuqbani M, Alhumimidi A, Baaboud A, Altamimi A (2024). Palliative care in Saudi Arabia: an updated assessment following the National Vision 2030 reforms. J Palliat Med.

[18] Hoffman BM, Zevon MA, D'Arrigo MC, Cecchini TB (2004). Screening for distress in cancer patients: the NCCN rapid-screening measure. Psychooncology.

[19] Renovanz M, Gutenberg A, Haug M (2013). Postsurgical screening for psychosocial disorders in neurooncological patients. Acta Neurochir (Wien).

[20] Alosaimi FD, Abdel-Aziz N, Alsaleh K, AlSheikh R, AlSheikh R, Abdel-Warith A (2018). Validity and feasibility of the Arabic version of distress thermometer for Saudi cancer patients. PLoS One.

[21] Zigmond AS, Snaith RP (1983). The hospital anxiety and depression scale. Acta Psychiatr Scand.

[22] Bjelland I, Dahl AA, Haug TT, Neckelmann D (2002). The validity of the Hospital Anxiety and Depression Scale: an updated literature review. J Psychosomat Res.

[23] Stafford L, Berk M, Jackson HJ (2007). Validity of the Hospital Anxiety and Depression Scale and Patient Health Questionnaire-9 to screen for depression in patients with coronary artery disease. Gen Hosp Psychiatry.

[24] Terkawi AS, Tsang S, AlKahtani GJ (2017). Development and validation of Arabic version of the Hospital Anxiety and Depression Scale. Saudi J Anaesth.

[25] Funk-Lawler R, Mundey KR (2020). Understanding distress among patients with cancer receiving specialized, supportive care services. Am J Hosp Palliat Care.

[26] Ascencio-Huertas L, Allende-Pérez SR, Pastrana T (2021). Associated factors of distress in patients with advanced cancer: a retrospective study. Palliat Support Care.

[27] Tergas AI, Razavi M, Loscalzo MJ, Rocha-Cadman X, Maciejewski PK, Prigerson HG, Clark L (2023). Patient characteristics associated with psychosocial distress about end-of-life. J Clin Oncol.

[28] Silva S, Bártolo A, Santos IM, Pereira A, Monteiro S (2022). Towards a better understanding of the factors associated with distress in elderly cancer patients: a systematic review. Int J Environ Res Public Health.

[29] Abu-Odah H, Molassiotis A, Zhao IY, Su JJ, Allsop MJ (2022). Psychological distress and associated factors among Palestinian advanced cancer patients: a cross-sectional study. Front Psychol.

[30] Ostwal SP, Singh R, Sanghavi PR, Patel H, Anandi Q (2021). Correlation between symptom burden and perceived distress in advanced head and neck cancer: a prospective observational study. Indian J Palliat Care.

[31] Zucca A, Sanson-Fisher R, Waller A, Carey M, Boyes AW, Proietto A (2016). Does screening for physical and psychosocial symptoms vary between medical oncology treatment centres?. Psychooncology.

[32] Goerling U, Hinz A, Koch-Gromus U, Hufeld JM, Esser P, Mehnert-Theuerkauf A (2023). Prevalence and severity of anxiety in cancer patients: results from a multi-center cohort study in Germany. J Cancer Res Clin Oncol.

[33] Velasco-Durantez V, Cruz-Castellanos P, Hernandez R (2024). Prospective study of predictors for anxiety, depression, and somatization in a sample of 1807 cancer patients. Sci Rep.

[34] Tang ST, Chen JS, Chou WC (2016). Longitudinal analysis of severe anxiety symptoms in the last year of life among patients with advanced cancer: relationships with proximity to death, burden, and social support. J Natl Compr Canc Netw.

[35] Bergerot CD, Razavi M, Clark KL, Philip EJ, Pal SK, Loscalzo M, Dale W (2021). Emotional problem-related distress screening and its prevalence by cancer type: assessment by patients' characteristics and level of assistance requested. Psychooncology.

[36] Poort H, Jacobs JM, Pirl WF, Temel JS, Greer JA (2020). Fatigue in patients on oral targeted or chemotherapy for cancer and associations with anxiety, depression, and quality of life. Palliat Support Care.

[37] Gontijo Garcia GS, Meira KC, de Souza AH, Guimarães NS (2023). Anxiety and depression disorders in oncological patients under palliative care at a hospital service: a cross-sectional study. BMC Palliat Care.

[38] Subramaniam DS, Zhang Z, Timmer Z, DeMarco EC, Poirier MP, Hinyard LJ (2024). Palliative care and mental health among pancreatic cancer patients in the United States: an examination of service utilization and health outcomes. Healthcare (Basel).

[39] Almalki AH, Alblowi M, Aljasser AS, Nafisah F, Alhedaithi I (2021). Anxiety and depression among palliative care cancer patients in Saudi Arabia: prevalence rates by age, gender, and cancer type. Int J Med Dev Ctries.

[40] Grotmol KS, Lie HC, Loge JH (2019). Patients with advanced cancer and depression report a significantly higher symptom burden than non-depressed patients. Palliat Support Care.

[41] Lee EM, Jiménez-Fonseca P, Hernández R (2023). The role of financial difficulties as a mediator between physical symptoms and depression in advanced cancer patients. Curr Oncol.

[42] Rodríguez-González A, Velasco-Durántez V, Cruz-Castellanos P (2023). Mental adjustment, functional status, and depression in advanced cancer patients. Int J Environ Res Public Health.

[43] Lobefaro R, Rota S, Porcu L (2022). Cancer-related fatigue and depression: a monocentric, prospective, cross-sectional study in advanced solid tumors. ESMO Open.

[44] Fisher HM, Winger JG, Miller SN (2021). Relationship between social support, physical symptoms, and depression in women with breast cancer and pain. Support Care Cancer.

[45] Gray NA, Kamal AH, Hanson LC, Bull J, Kutner JS, Ritchie CS, Johnson KS (2022). Clinician perspectives guiding approach to comprehensiveness of palliative care assessment. J Palliat Med.

[46] Graboyes EM, Chaiyachati KH, Sisto Gall J (2022). Addressing transportation insecurity among patients with cancer. J Natl Cancer Inst.

[47] Wercholuk AN, Parikh AA, Snyder RA (2022). The road less traveled: transportation barriers to cancer care delivery in the rural patient population. JCO Oncol Pract.

[48] Arch JJ, Bright EE, Finkelstein LB, Fink RM, Mitchell JL, Andorsky DJ, Kutner JS (2023). Anxiety and depression in metastatic cancer: a critical review of negative impacts on advance care planning and end-of-life decision making with practical recommendations. JCO Oncol Pract.

[49] Hartl DM, Ferlito A, Brasnu DF, Langendijk JA, Rinaldo A, Silver CE, Wolf GT (2011). Evidence-based review of treatment options for patients with glottic cancer. Head Neck.

[50] Chou WS, Hamel LM, Thai CL (2017). Discussing prognosis and treatment goals with patients with advanced cancer: a qualitative analysis of oncologists' language. Health Expect.

[51] Kazer MW, Bailey DE Jr, Chipman J (2013). Uncertainty and perception of danger among patients undergoing treatment for prostate cancer. BJU Int.

[52] Brom L, De Snoo-Trimp JC, Onwuteaka-Philipsen BD, Widdershoven GA, Stiggelbout AM, Pasman HR (2017). Challenges in shared decision making in advanced cancer care: a qualitative longitudinal observational and interview study. Health Expect.

[53] Hasdenteufel M, Quintard B (2022). Dyadic experiences and psychosocial management of couples facing advanced cancer: a systematic review of the literature. Front Psychol.

[54] van Roij J, Raijmakers N, Johnsen AT, Hansen MB, Thijs-Visser M, van de Poll-Franse L (2022). Sexual health and closeness in couples coping with advanced cancer: results of a multicenter observational study (eQuiPe). Palliat Med.

[55] Laryionava K, Winkler EC (2021). Dealing with family conflicts in decision-making in end-of-life care of advanced cancer patients. Curr Oncol Rep.

[56] Hamano J, Masukawa K, Tsuneto S, Shima Y, Morita T, Kizawa Y, Miyashita M (2022). Are family relationships associated with family conflict in advanced cancer patients?. Psychooncology.

[57] Kissane DW, Zaider TI, Li Y (2016). Randomized controlled trial of family therapy in advanced cancer continued into bereavement. J Clin Oncol.

[58] Chopra D, De La Garza R (2019). Depressive, anxiety, and distress symptoms among cancer patients who endorse appearance problems. Palliat Support Care.

[59] Brose JM, Willis E, Morgan DD (2023). The intentional pursuit of everyday life while dying: a longitudinal qualitative study of working-aged adults living with advanced cancer. Palliat Med.

[60] Shimoda K, Imai H, Tsuji T, Tsuchiya K, Tajima H, Kanemaki H, Tozato F (2019). Factors affecting the performance of activities of daily living in patients with advanced cancer undergoing inpatient rehabilitation: results from a retrospective observational study. J Phys Ther Sci.

[61] Amano K, Morita T, Miura T (2023). Development and validation of questionnaires for eating-related distress among advanced cancer patients and families. J Cachexia Sarcopenia Muscle.

[62] Singhal S, Qin Z, Peterson DR (2023). Association of nutrition with psychological health and quality of life among older adults with advanced cancer. J Clin Oncol.

[63] Strasser F (2006). Eating-related distress of patients with advanced, incurable cancer and of their partners. Cachexia and Wasting: A Modern Approach.

[64] Molassiotis A, Brown T, Cheng HL (2021). The effects of a family-centered psychosocial-based nutrition intervention in patients with advanced cancer: the PiCNIC2 pilot randomised controlled trial. Nutr J.

[65] Tennison JM, Pally A, Fellman BM, Westney OL, Bruera E (2023). Urinary dysfunction: frequency, risk factors, and interventions in patients with cancer during acute inpatient rehabilitation. J Cancer.

[66] Mercadante S, Ferrera P, Casuccio A (2011). Prevalence of opioid-related dysuria in patients with advanced cancer having pain. Am J Hosp Palliat Care.

[67] Cho OH, Yoo YS, Kim JC, Park RH, Hwang KH (2018). Factors influencing lower urinary tract symptoms in advanced cancer patients with chemotherapy-induced peripheral neuropathy. Int Neurourol J.

[68] Kristensen MH, Elfeki H, Sinimäki S, Laurberg S, Emmertsen KJ (2021). Urinary dysfunction after colorectal cancer treatment and impact on quality of life-a national cross-sectional study in males. Colorectal Dis.

[69] Tsushima T, Miura T, Hachiya T (2019). Treatment recommendations for urological symptoms in cancer patients: clinical guidelines from the Japanese Society for Palliative Medicine. J Palliat Med.

